# KiGGS Wave 2 longitudinal component – data collection design and developments in the numbers of participants in the KiGGS cohort

**DOI:** 10.17886/RKI-GBE-2018-035

**Published:** 2018-03-15

**Authors:** Michael Lange*, Robert Hoffmann, Elvira Mauz, Robin Houben, Antje Gößwald, Angelika Schaffrath Rosario, Bärbel-Maria Kurth

**Affiliations:** Robert Koch Institute, Berlin, Department of Epidemiology and Health Monitoring

**Keywords:** KIGGS COHORT, LONGITUDINAL COMPONENT, REPEAT PARTICIPATION, CHILDREN AND ADOLESCENTS, HEALTH MONITORING

## Abstract

The German Health Interview and Examination Survey for Children and Adolescents (KiGGS) is conducted within the health monitoring framework that has been established at the Robert Koch Institute (RKI). In addition to regular cross-sectional studies of the current health of children and adolescents living in Germany, KiGGS also includes a longitudinal component – the KiGGS cohort. The longitudinal data, which can be linked individually throughout the various waves of the study, enables developments in health and their associated influencing factors to be analysed during the life course. Participants from the KiGGS baseline study form the baseline of the KiGGS cohort. The baseline study was carried out between 2003 and 2006 as a nationwide interview and examination survey of children and adolescents aged between 0 and 17 years. The KiGGS cohort comprises the 17,641 participants who, after taking part in the baseline study, also agreed to participate in recurring follow-ups that are to continue through adolescence into adulthood. Until now, two follow-up studies have been conducted: KiGGS Wave 1 (2009-2012, n=11,992) and KiGGS Wave 2 (2014-2017), which, in line with the baseline study, was conducted as an interview and examination survey. A total of 10,853 repeat participants were interviewed for KiGGS Wave 2; 6,465 people also took part in an examination. As such, 61.3% of the people who originally participated in the baseline study also provided data from interviews for KiGGS Wave 2. In addition, 50.8% have provided various forms of data for all three of the survey’s waves. This data pool can help answer numerous questions from the epidemiological life course discipline regarding the population living in Germany; at the time of the baseline study, these participants were children and adolescents. In order to exploit the full potential of the study for life course research and to be able to trace the health and social development of different generations in the future, the concepts on which the study is based are to be further developed, and innovative strategies for participant retention are to be drawn up.

## 1. Background

### 1.1 The KiGGS cohort at the Robert Koch Institute

The Robert Koch Institute (RKI) is a German public health institute that has been tasked with monitoring developments in the health of the population living in Germany as part of the health monitoring framework [1-3]. The German Health Interview and Examination Survey for Children and Adolescents (KiGGS) is conducted at regular intervals and acts as a central source of information that collects wide-ranging, reliable data on child and adolescent health. KiGGS comprises a cross-sectional and a longitudinal component, each of which has different objectives [4, 5].

**KiGGS Wave 2**

Second follow-up to the German Health Interview and Examination Survey for Children and Adolescents

**Data owner:** Robert Koch Institute

**Aim:** Providing reliable information on health status, health-related behaviour, living conditions, protective and risk factors, and health care among children, adolescents and young adults living in Germany, with the possibility of trend and longitudinal analyses

**Study design**: Combined cross-sectional and cohort study

**Cross-sectional study in KiGGS Wave 2**

**Age range:** 0-17 years

**Population:** Children and adolescents with permanent residence in Germany

**Sampling:** Samples from official residency registries - randomly selected children and adolescents from the 167 cities and municipalities covered by the KiGGS baseline study

**Sample size:** 15,023 participants

**KiGGS cohort study in KiGGS Wave 2**

**Age range:** 10-31 years

**Sampling:** Re-invitation of everyone who took part in the KiGGS baseline study and who was willing to participate in a follow-up

**Sample size:** 10,853 participants

**KiGGS survey waves**

► KiGGS baseline study (2003-2006), examination and interview survey
► KiGGS Wave 1 (2009-2012), interview survey
► KiGGS Wave 2 (2014-2017), examination and interview survey

More information is available at www.kiggs-studie.de/english

The cross-sectional component comprises the recurrent collection of representative data on the health status of children and adolescents in Germany aged between 0 and 17 years. Until now, three nationally representative studies of the children and adolescents living in Germany during each study period have been conducted: the KiGGS baseline study (2003-2006), KiGGS Wave 1 (2009-2012) [6] and KiGGS Wave 2 (2014-2017) [7]. The data gathered for these studies provides a profound basis with which to calculate prevalences and conduct context analyses for each period while identifying changes over time (trends) among children and adolescents aged between 0 and 17 years in Germany [4, 8].

**Figure 1:**
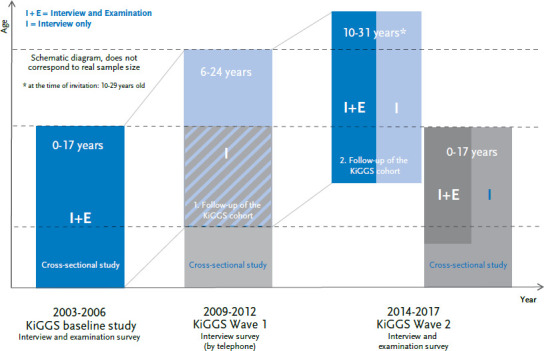
The study design of the KiGGS cohort Source: Adapted from Mauz et al. [7]

The KiGGS cohort is the longitudinal component of the study, and it involves the repeated collection of data of KiGGS baseline participants into adulthood. To date, two follow-up studies have been conducted of the KiGGS cohort: KiGGS Wave 1 [9] and KiGGS Wave 2 [7] (Figure 1). In both cases, data was also gathered for the respective cross-sectional component of the KiGGS survey at the same time as the follow-ups were conducted. The approach was aimed at ensuring economic synergies. This includes the shared use of study infrastructure for participant recruitment, data collection and analysis. All previous studies undertaken with the KiGGS cohort have been supplemented by two independent longitudinal modular studies. The BELLA mental health module and the MoMo motor performance and physical activity module are issue-based, in-depth studies and the data gathered for them can be linked to data collected for the KiGGS cohort.

### 1.2 The KiGGS baseline study and its follow-up studies

The baseline sample of the KiGGS cohort comprises all of the 17,641 children and adolescents who participated in the representative KiGGS baseline study (age range: 0 to 17 years). The baseline study was carried out between 2003 and 2006 as an examination and interview survey at 167 sample points [10]. The two-stage sampling procedure is described in detail elsewhere [8, 10, 11]. Data collection was conducted in temporary examination centres set up for this purpose. Medical measurements and tests were conducted and blood and urine samples were taken as part of the examinations [10]. In retrospect, one participant out of 17,641 asked to have their contact and study data deleted. Content related analysis, therefore, is based on data gathered from 17,640 participants.

Those who provided their consent were invited to participate in the follow-ups (KiGGS Wave 1 and KiGGS Wave 2). In order to reliably identify developments in health during the life course and their associated influencing factors, it is essential that as many of the participants from the baseline study as possible participate in subsequent study waves. Furthermore, it is important to avoid systematic bias as far as possible, with regard to repeat participation in order to deliver valid results.

**Figure 2:**
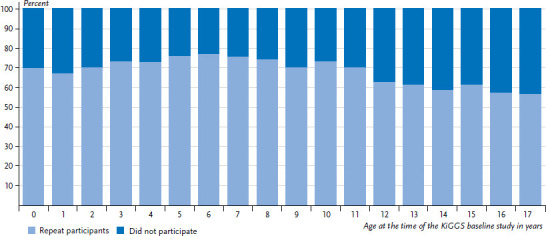
Repeat participation in the first telephone interview follow-up survey KiGGS Wave 1 according to age at the time of the baseline study (n=8,655 girls; n=8,986 boys) Source: KiGGS Wave 1 (KiGGS cohort)

The first follow-up survey – KiGGS Wave 1 – was conducted between 2009 and 2012 as a computer-assisted telephone interview survey with a reduced range of topics [6]. A total of 11,992 (6,078 females, 5,914 males) of the people who had participated in the KiGGS baseline study repeatedly participated. There was a slightly reduced willingness to participate in KiGGS Wave 1 among adult-aged members of the cohort (Figure 2).

In addition to age, factors such as male gender, coming from a family with migration background or a single parent family, as well as low socioeconomic status were associated with lower willingness to participate in KiGGS Wave 1. Longitudinal weighting was employed in KiGGS Wave 1 in order to compensate for bias in the cohort, as far as possible (see Section 3.4).

### 1.3 The aims of this article

This article describes the methodology applied in the second KiGGS follow-up survey – KiGGS Wave 2. It will be described for which subgroups data from interviews and/or examinations are available. Furthermore, this article explains the implemented measures to ensure that data for KiGGS Wave 2 could be gathered from as many baseline study participants as possible. The results section also states the number of people (who were children and adolescents at the time of the baseline study) who were recruited to Wave 2 despite having declined to participate in KiGGS Wave 1. After a brief discussion of weighting factors, this article closes with a summary and an outlook.

## 2. The methodology employed in KiGGS Wave 2

### 2.1 The initial KiGGS cohort sample

Work with the KiGGS cohort during KiGGS Wave 2 began with a focus on the 17,641 people who had participated in the KiGGS baseline study (as was the case with KiGGS Wave 1, see Section 1.2). KiGGS Wave 2 aimed to recruit as many of the study’s initial participants – hereafter referred to as study participants – regardless as to whether they had participated in KiGGS Wave 1. Invitations were not sent out to study participants if it was clear that they had moved abroad, had died, if it had not been possible to locate them for Wave 1 or if they had asked not be contacted in the future. Among the latter, after the baseline study had been completed, one study participant requested the retrospective deletion of all contact and survey data relating to their person. Two study participants from the cohort had changed their information on gender from female to male by the time KiGGS Wave 2 was conducted.

### 2.2 Planning the study

In line with the baseline study, KiGGS Wave 2 was carried out as an examination and interview survey. However, it covered a broader range of topics [7]. The study design for KiGGS Wave 2 was developed with the aim of recruiting as many study participants from the KiGGS baseline study as possible and ensuring that they provided interview data at the very least. In addition, measures were undertaken to collect examination data from as many people as possible. Whereas the interviews could be conducted by sending out self-administered paper questionnaires to the study participants wherever they lived, the examination programme relied on a specific spatial infrastructure (attendance at an examination centre). The high level of mobility among the now adult-aged cohort members meant that potential study participants were no longer merely distributed between 167 sample points but across almost 2,000 different municipalities. As KiGGS Wave 2 involved the simultaneous conduction of both an up-to-date cross-sectional study and the second follow-up of the cohort study, temporary examination centres were established in the original 167 sample points out of economic and logistical reasons for both the cross-sectional component of KiGGS [8] and the KiGGS cohort (see Section 2.3). Both study participants from the cross-sectional sample and cohort members who still lived in their original places of residence were invited to an examination centre. The current place of residence of the people from the baseline sample was determined immediately prior to the invitation being sent out using information gained from municipal population registries. This led the study participants to be divided into two groups: a group of potential study participants who still lived in the original sample point (and therefore were to be examined and interviewed), and a group comprising all of the other study participants (who were therefore only to be interviewed). The invitations were then sent by post to anyone who had not stated that they no longer wished to be contacted (see Section 2.1).

### 2.3 Data collection

Collection of the longitudinal data for KiGGS Wave 2 was conducted between September 2014 and August 2017. The examination programme was carried out at the nationwide level by three examination staff teams working in parallel; this enabled examinations to be undertaken in three places at the same time. A so-called road map was devised at the beginning of the study which set out the order in which the sample points were to be visited [8]. All potential participants received self-administered paper questionnaires by post. In the case of young children, the parents or legal guardians were asked to provide information about their child’s health. Children and adolescents aged 10 years or above were asked to answer the questionnaires by themselves. A questionnaire on nutrition was also sent out, and this was also to be answered by the children and adolescents. Potential study participants who had now reached adult-age received two questionnaires to be filled out by themselves: one on health and one on nutrition. Study participants who did not attend their examination were sent a questionnaire by mail on physician-diagnosed conditions to replace the personal medical interview that would have been conducted at the examination centre.

### 2.4 Invitations and participation

**Figure 3:**
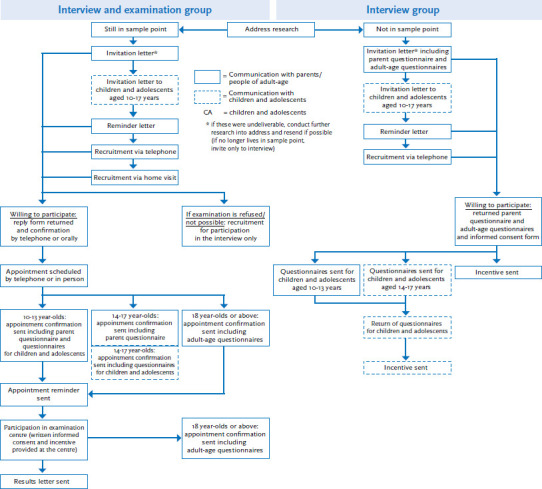
Study participant recruitment for the cohort in KiGGS Wave 2 Source: Own diagram

The invitation to participate in an examination and interview or an interview was sent out by post in accordance with the road map. Invitations were usually sent out six weeks before the examination centre opened at each sample point (Figure 3). When the invitations to KiGGS Wave 2 were sent out, the people who had participated in the KiGGS baseline study were already between 10 and 29 years old (by the time they participated, some of them were already 31 years old). In the case of minors, their parents or legal guardians were contacted (simply called parents below). Legal and ethical reasons meant that the parents acted as the central point of contact for all survey matters. Prospective study participants aged 18 or older were contacted directly. The letters of invitation contained a booklet detailing information about the current study wave. About three days after the invitation letter, children and adolescents aged 10 or above received a letter of invitation specifically addressed to them, accompanied by an age-appropriate information sheet [8].

The invitation was followed by three further recruitment stages, as was the case with the cross-sectional survey, aiming to reach those parents and adult-aged study participants who had yet to answer. First, a reminder was sent out about ten days after the initial invitation. Second, an attempt was made to contact the parents or adult-aged study participants by telephone. Usually at least one, if not more, telephone numbers were available for each of the study participants from the KiGGS baseline study and from the telephone interviews conducted for KiGGS Wave 1. Due to limited staff and financial resources, however, phone calls – which necessitate a lot of time and personnel – were initially only carried out to the full extent for people who had been invited to an examination. This focus was necessary because the temporary nature of the examination centres essentially required to hold to the time slots allocated within the road map. Less exhaustive attempts were initially made to contact study participants who had only been invited for an interview; efforts were increased at a later date. Third, home visits were conducted to recruit the parents and now adult-aged study participants who were still living in the same sample point and who had been invited to an examination. These home visits were conducted one week prior to the examination centre opening. In general, telephone and personal conversations are a useful means of clearing individual reservations, balancing information deficits and building trust in the study’s goals. By contacting cohort members directly, emphasis could be put on the importance of previous participation in the study and the value of repeated participation. Specially trained staff was employed in these recruitment measures. A more detailed description of the procedure is available in the article KiGGS Wave 2 cross-sectional study – participant acquisition, response rates and representativeness [8].

### 2.5 Further measures to improve participation

A variety of measures were implemented to improve participation in the study both in terms of numbers and participant composition. Some of the measures concern information management, providing incentives (such as allowances) or reducing barriers to participation and managing appointments in the examination component [8]. Measures to improve the participation of people with migration background are described in detail in the article Improving the inclusion and participation of children and adolescents with a migration background in KiGGS Wave 2 [12]. For example, the examination staff teams and the staff responsible for telephone or home visit recruitment took part in intercultural skill trainings. While some measures were also employed in the cross-sectional component, the longitudinal component of KiGGS Wave 2 also included the following specifically designed measures:

► **Additional research was conducted to find out where people lived when invitations were returned as undeliverable:** Invitations were resent if “Deutsche Post” provided a new address for people whose invitations were returned as undeliverable. If the new address was outside of the original sample point, the person was only invited to an interview. On the other hand, if an invitation was returned without a new address, existing telephone numbers were used to contact the study participants and ask for a valid address. If it was only possible to reach the parents of adult-aged study participants, the parents were asked to inform their children about the study and to ask them to contact the RKI. If no or no valid telephone number was available, the same request was sent out in writing to parents whose addresses - known from the baseline study or from KiGGS Wave 1 - differed from that of their child.
► **Recruitment for interviews in cases where people opted not to take part in an examination:** If participation in an examination was either not possible or if a parent or adult-aged study participant declined to (have their child) take part, they were asked to participate in an interview. This was subject to that the reason why they declined to take part in an examination did not argue against the participation in an interview. This happened both during the various phases of recruitment (see Section 2.4) and in cases of non-attendances at an examination appointment. As such, some study participants were invited to participate in an examination, but only participated in the interview.

In addition, several measures were implemented on a one-off basis (as such, they are not included in Figure 3) in order to increase the number of participants among adult-aged members of the cohort. Among others, these measures included:

► **Additional examination slot in Berlin:** An unscheduled examination centre was established in Berlin during the last road map slot. Adult-aged study participants who lived in Berlin or had moved there from another sample point and who had not been reached until that time were contacted again and invited to participate in an examination. This includes adult-aged study participants who had initially been invited to an examination and those who had only been invited to an interview.
► **Additional online questionnaire:** Study participants who had not been reached by mid-May 2017 and were currently of adult-age were invited again by post. They were provided with the opportunity to answer an online questionnaire. This invitation was sent out on the assumption that adults who had not participated in an examination and/or self-administered paper questionnaire sent out by post might be more willing to take part in an online questionnaire. In order to increase the attractiveness of participating, the interview programme was shortened and only included the questionnaire on health (no information was sought from the questionnaire on nutrition). In addition, a higher allowance was offered. This questionnaire was sent out to adults who had initially been invited to an examination and those who had only originally been invited to an interview.

### 2.6 Data protection and ethical considerations

KiGGS Wave 2 is subject to strict compliance with the data protection provisions set out in the Federal Data Protection Act. Hannover Medical School’s ethics committee examined and approved the ethics of the study (No. 2275-2014). The Federal Commissioner for Data Protection and Freedom of Information in Germany received the KiGGS Wave 2 study concept and had no objections. Together with the invitation to the survey, participants, their parents and/or legal guardians were informed about those responsible for the survey, the objectives and content of the survey, voluntary participation and data protection. They provided their informed consent in writing.

## 3. Participation in KiGGS Wave 2 and the number of study participants who participated in the cohort over time

### 3.1 Participation in KiGGS Wave 2

A total of 13,085 study participants (6,203 female, 6,882 male) were assigned to a group envisaged for an examination and interview; 4,556 study participants (2,451 female, 2,105 male) were assigned to a group that was only invited to an interview.

In total, 10,853 of the 17,641 people who participated in the KiGGS baseline study also took part in KiGGS Wave 2. Data from interviews are available from all of these study participants. Thus, a total of 61.5% of the study participants who took part in the baseline study participated in the second follow-up. A greater number of female study parti cipants (66.9%) repeatedly participated in KiGGS than males (56.3%). Additional examination data are available for 6,465 of these individuals. This amounts to 36.6% of the baseline sample. The most important reason that examination data is not available from a larger number of study participants is related to the study design: study participants were only invited to an examination if they lived in the same sample point as during the baseline study. 13,084 study participants were assigned to an examination, and 49.4% took part. Once again, participation was higher among females (52.5%) than males (46.7%).

**Figure 4:**
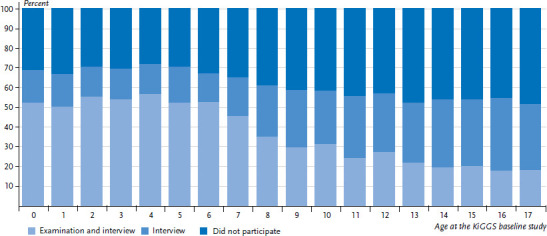
Repeat participation by study participants from the baseline sample in examinations and interviews or only interviews for KiGGS Wave 2 according to age at the time of the baseline study (n=8,654 female, n=8,987 male) Source: KiGGS Wave 2 (KiGGS cohort)

If the data is viewed according to age (the study participants were aged between 0 and 17 years when they participated in the baseline study), it becomes clear that a lot less examination data are available for people who are now adults (Figure 4). This can be explained by their reduced willingness to participate and by the fact that a higher proportion of older study participants no longer lives in the original sample point and were therefore not invited to an examination (data not shown).

### 3.2 Developments in the numbers of study participants across all study waves

**Figure 5:**
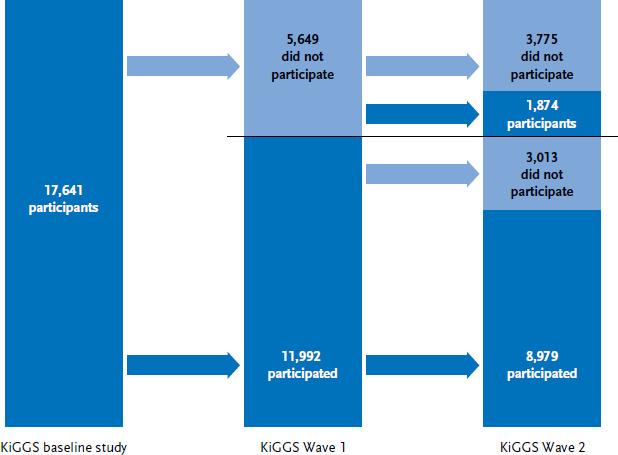
Participation of the baseline sample in the various KiGGS study waves Source: KiGGS baseline study (2003-2006), KiGGS Wave 1 (2009-2012), KiGGS Wave 2 (2014-2017)

Of the 17,641 people who took part in the KiGGS baseline study, 8,979 people (4,796 female, 4,183 male), in other words, 50.9% of the baseline sample, provided data for all three study waves (Figure 5). 1,874 people (994 female, 880 male) who participated in KiGGS Wave 2 did not take part in KiGGS Wave 1 (this corresponds to 10.6% of the baseline sample). 3,013 people (1,732 female, 1,281 male), in other words 17.1%, provided data for the baseline study and Wave 1, but did not do so for Wave 2. In total, 3,775 study participants from the baseline study (1,583 female, 2,192 male) could not be recruited for either of the two follow-up surveys. This corresponds to 21.4% of the people who participated in the baseline study.

### 3.3 Repeat participation

During each wave of the study, parents and adult-aged study participants were asked whether they would consent to being invited to participate again in the future. By the end of the second KiGGS Wave 4% of the original sample had declined to be contacted again, meaning that these individuals cannot be invited to any future follow-up.

### 3.4 Weighting

As described in Section 3.1, 61.5% of the people who took part in the KiGGS baseline study participated in KiGGS Wave 2. Willingness to participate in further study waves can vary between different groups of the study population, leading to bias in longitudinal analyses. Weighting factors are calculated in order to compensate for this problem. This involves employing a logistic regression model to predict the likelihood of non-participation using socio-demo-graphic and health-related indicators developed from the KiGGS baseline study. This results in a higher weighting for groups that tend to be less willing to participate. Three separate weighting factors were calculated: a) for the entire study population (with data from interviews), b) for study participants who took part in an examination and interview, and c) for the subgroup of study participants with laboratory analyses. The weighting factors were developed primarily using socio-demographic characteristics such as age, socioeconomic status of the family of origin, education, migration background, size of the residential district, as well as the participant’s mother’s smoking behaviour.

## 4. Summary, discussion and outlook

The KiGGS baseline study was the first national population-based study of the health of children and adolescents in Germany. It provides wide-ranging data on the health and social situation of 0 to 17 year-old children and adolescents in Germany. The 17,641 people who participated in the baseline study deliver a solid basis for further study waves of the KiGGS cohort.

Now that KiGGS Wave 2 has been completed, data from two further study waves are available from the people who participated in the baseline study. This longitudinal data, which can be individually linked across the survey’s waves, means that it is possible to observe and analyse health and social developments into adulthood among these individuals who were children and adolescents at the time of the baseline study. The oldest study participants are now 31 years old.

61.5% of the people who participated in the baseline study took part in the interview component of KiGGS Wave 2. Therefore, wide-ranging data from interviews have been gathered from a total of 10,853 participants. This means that KiGGS Wave 2 reached a similarly large proportion of participants from the baseline study as the telephone-based survey conducted in KiGGS Wave 1. Although the range of questions employed during Wave 1 was continued, it was also partially broadened and covered the topics of health, health-related behaviour and social life situation [7]. The data provides the opportunity to answer numerous questions from epidemiological life course disciplines for the population living in Germany, which, at the time of the baseline study, was still in childhood and adolescence.

Nevertheless, a significantly lower proportion (36.6%) of the baseline study participated in an examination and an interview for KiGGS Wave 2. This was partly due to the fact that examinations were only carried out in the same sample points that were used during the baseline study. Thus, people could only be invited to an examination if they still lived in the same sample point as they did during the baseline study. Of those who were assigned to an examination, 49.4% took part in the examination programme. Consequently, examination data are available for two observation periods for a total of 6,465 study participants. These data are supplemented by wide-ranging data from interviews which can be used to conduct differentiated longitudinal analyses. The first descriptive course analyses that have been published cover obesity and allergic sensitisation.

Unfortunately, much less examination data are avail able for adults compared to minor-aged study participants. On the one hand, this is because adults are particularly mobile compared to young people and, therefore, could not always be invited to examinations. However, they were also less willing to participate in examinations. Detailed analyses of these aspects are planned. A lower level of parti cipation may affect the validity of longitudinal analyses if groups differ significantly in their likelihood of parti cipating again. Nevertheless, such differences can be compensated for by weighting, provided that they can be explained by survey variables covered by previous study waves.

There certainly is a good basis for continuing the KiGGS cohort. Despite the large intervals between follow-ups, many study participants are apparently strongly committed to KiGGS. This is clear from the fact that about two-thirds of the baseline study took part in Wave 2 and that during the entire study period only 4% of study participants or their parents decided to cut ties with the study. The fact that 33% of the cohort who did not participate in KiGGS Wave 1 were ‘won back’ for Wave 2 was a welcome development. Nevertheless, 21% of the baseline sample neither participated in Wave 1 nor Wave 2. This includes study participants who were no longer invited (because they no longer wished to be contacted, had died or moved abroad) as well as people who were invited, but declined to participate. Further analysis is needed on this group’s composition, of whether some of these study participants could indeed be recruited in the future, and, if so, which mea sures would be needed to do so.

The adult-aged study participants will continue to be of particular importance in future KiGGS waves. More than half of the participants in the KiGGS cohort have already reached adult-age. Moreover, the entire cohort will have reached adulthood by 2024. Thus, the KiGGS cohort will gain a significance of its own and will no longer merely be associated with monitoring the health of children and adolescents. In view of the great potential that the cohort provides for innovation and research, new concepts for survey content and methods as well as for the analysis of ever more complex datasets will have to be developed. The first ideas have already been considered and need to be tested, discussed, evaluated, published and implemented.

From the point of view of public health, the KiGGS cohort constitutes a valuable resource for developing evidence-based measures that can improve the health of the population, beginning in childhood and adolescence. In terms of its representative basis (for children and adolescents living in Germany at the beginning of the 21st century), the size of the sample and the breadth of its data means that the KiGGS cohort continues to be unique in Germany.

## Key statements

- Developments in the health of the participants from the KiGGS baseline study are followed into adolescence and adulthood in the KiGGS cohort.
- The cohort study provides longitudinal data that enables analyses to be made of developmental trajectories and their influencing factors.
- Until now, two KiGGS follow-up studies have been conducted: KiGGS Wave 1 and KiGGS Wave 2.
- A total of 11,992 repeat participants were interviewed for KiGGS Wave 1.
- A total of 10,853 repeat participants were interviewed for KiGGS Wave 2; 6,465 participated in an examination.

## Figures and Tables

**Table 1:** Overview of cohort participants in KiGGS Wave 2 Source: KiGGS baseline study (2003-2006), KiGGS Wave 2 (2014-2017)

	Repeat participants with interview data			Subgroup of repeat participants with additional examination data		
	Female*	Male*	Total	Female*	Male*	Total
Baseline sample (participants in the KiGGS baseline study)	8,655	8,986	17,641	8,654	8,987	17,641
Participated in KiGGS Wave 2	5,790	5,063	10,853	3,254	3,211	6,465
Proportion of KiGGS Wave 2 participants in the baseline sample	66.9%	56.3%	61.5%	37.6%	35.7%	36.6%

* Details on gender provided for KiGGS Wave 2
